# Bisphenol a exposure in Mexico City and risk of prematurity: a pilot nested case control study

**DOI:** 10.1186/1476-069X-9-62

**Published:** 2010-10-18

**Authors:** David Cantonwine, John D Meeker, Howard Hu, Brisa N Sánchez, Héctor Lamadrid-Figueroa, Adriana Mercado-García, Gamola Z Fortenberry, Antonia M Calafat, Martha Maria Téllez-Rojo

**Affiliations:** 1 Department of Environmental Health Sciences, University of Michigan School of Public Health, Ann Arbor, Michigan, USA; 2 Department of Environmental Health, Harvard School of Public Health, Boston, Massachusetts, USA; 3 Channing Laboratory, Department of Medicine, Brigham and Women's Hospital, Harvard Medical School, Boston, Massachusetts, USA; 4 Department of Biostatistics, University of Michigan School of Public Health, Ann Arbor, Michigan, USA; 5 Division of Statistics, Center for Evaluation Research and Surveys, National Institute of Public Health, Mexico, Cuernavaca, Morelos, México; 6 Division of Environmental Health, Center for Population Health Research, National Institute of Public Health, Cuernavaca, Morelos, México; 7 Centers for Disease and Control and Prevention, Atlanta, GA, USA

## Abstract

**Background:**

Presence of Bisphenol A (BPA) has been documented worldwide in a variety of human biological samples. There is growing evidence that low level BPA exposure may impact placental tissue development and thyroid function in humans. The aim of this present pilot study was to determine urinary concentrations of BPA during the last trimester of pregnancy among a small subset of women in Mexico City, Mexico and relate these concentrations to risk of delivering prematurely.

**Methods:**

A nested case-control subset of 60 participants in the Early Life Exposure in Mexico to ENvironmental Toxicants (ELEMENT) study in Mexico City, Mexico were selected based on delivering less than or equal to 37 weeks of gestation and greater than 37 weeks of gestation. Third trimester archived spot urine samples were analyzed by online solid phase extraction coupled with high performance liquid chromatography isotope dilution tandem mass spectrometry.

**Results:**

BPA was detected in 80.0% (N = 48) of the urine samples; total concentrations ranged from < 0.4 μg/L to 6.7 μg/L; uncorrected geometric mean was 1.52 μg/L. The adjusted odds ratio of delivering less than or equal to 37 weeks in relation to specific gravity adjusted third trimester BPA concentration was 1.91 (95%CI 0.93, 3.91, p-value = 0.08). When cases were further restricted to births occurring prior to the 37^th ^week (n = 12), the odds ratio for specific-gravity adjusted BPA was larger and statistically significant (p < 0.05).

**Conclusions:**

This is the first study to document measurable levels of BPA in the urine of a population of Mexican women. This study also provides preliminary evidence, based on a single spot urine sample collected during the third trimester, that pregnant women who delivered less than or equal to 37 weeks of gestation and prematurely (< 37 weeks) had higher urinary concentrations of BPA compared to women delivering after 37 weeks.

## Background

Bisphenol A (BPA, CAS no. 80-05-7) is produced in high volume worldwide for use in a variety of industrial and consumer products, such as epoxy resins used to line food cans [1], polyester-styrene [2], and polycarbonate plastics which make up some baby bottles and other containers [3]. Due to this wide spread usage, the primary exposure route in humans is thought to occur via ingestion of food or water [4,5]. Calafat et al. documented extensive exposure to the general US population in a subset of the 2003-2004 National Health and Nutrition Examination Survey (NHANES), where 93% of the 2,517 participants had detectable BPA in their urine [6]. Additionally, other studies in the Netherlands [7], Germany [8], China [9], Japan [10], and Norway [11] have all found similar population wide exposure to BPA in each respective country. Human studies have also measured BPA in a variety of other human body fluids and some tissues. Of potential concern to fetal and infant health endpoints is the presence of BPA in follicular and amniotic fluid [12], umbilical cord blood and placental tissue [13], and breast milk [14].

BPA has been shown to have weak oestrogenic activity and potentially impacts thyroid function [15,16]. Additionally, recent evidence from in vitro studies suggests that fetal exposure to BPA can occur through placental exchange [17] and that BPA exposure at doses (0.02 - 0.1 μg/mL) close to those found in some pregnant women can induce cell death in isolated human cytotrophoblast cells [18]. In animal studies, environmentally-relevant concentrations of BPA have been associated with low birth weight [19]. By contrast, there is no evidence for this relationship or for a relationship with gestational length in humans [20,21].

The primary aim of this pilot study was to determine urinary concentrations of BPA during the last trimester of pregnancy among 60 women, a subset of participants in the Early Life Exposure in Mexico to ENvironmental Toxicants (ELEMENT) study in Mexico City, Mexico. A secondary aim of this study was to determine differences in BPA concentrations between women who delivered less than or equal to 37 weeks of gestation and after the completion of 37 weeks of gestation. To our knowledge, this is the first report on the biological monitoring of BPA in Mexico.

## Methods

### Sample Population

The present study was nested within a Mexican birth cohort study in which women were recruited during prenatal visits at one of four clinics of the Mexican Institute of Social Security in Mexico City between 2001 and 2003 and has been described elsewhere [22]. The research protocol was approved by the Ethics and Research Committees of all participating institutions. The involvement of the Centers for Disease Control and Prevention (CDC) laboratory was limited and determined not to constitute engagement in human subjects research. The study was described in detail to all participating mothers and all study participants gave informed consent.

Of the 1,853 women approached, 670 (36%) agreed to participate in the cohort study. Of these, archived third trimester urine samples, collected during 2001-2003, were available for 518 nonsmoking women because urine collection did not commence until after the initiation of study recruitment had begun. In the present nested case-control study conducted within these 518 women, 30 participants were selected randomly among women who had completed 38 or more weeks of gestation at the time of delivery. Additionally, 30 participants were selected among women who had delivered before 38 weeks of gestation at time of delivery, which included 12 women who delivered prior to 37 weeks.

### Measures of Bisphenol-A in Urine

A spot (second morning void) urine sample was collected from each woman during a third trimester visit (earliest urine collection occurred during the 30^th ^week of gestation) to the project's research center and frozen at -80°C. Samples were shipped on dry ice overnight to the CDC, where the total urinary concentration of BPA (free plus conjugated species) was measured using online solid-phase extraction (SPE) coupled to isotope dilution-high performance liquid chromatography (HPLC) - tandem mass spectrometry (MS/MS) on a system constructed from several HPLC Agilent 1100 modules (Agilent Technologies, Wilmington, DE) coupled to a triple quadrupole API 4000 mass spectrometer (Applied Biosystems, Foster City, CA). First, 100 μL of urine was treated with β-glucuronidase/sulfatase (*Helix pomatia*, H1; Sigma Chemical Co., St. Louis, MO) to hydrolyze the BPA-conjugated species. BPA was then retained and concentrated on a C18 reversed-phase size-exclusion SPE column (Merck KGaA, Germany), separated from other urine matrix components using a pair of monolithic HPLC columns (Merck KGaA), and detected by negative ion-atmospheric pressure chemical ionization-MS/MS. Low-concentration (~ 4 μg/L) and high-concentration (~ 20 μg/L) quality control materials, prepared with pooled human urine, were analyzed with analytical standards, reagent blanks, and unknown samples (15). Specific gravity was measured at the University of Michigan using a handheld digital refractometer (ATAGO Company Ltd., Tokyo, Japan). BPA concentrations were also corrected by creatinine (Cr; BPA concentrations expressed as μg/g creatinine), which was measured at the University of Michigan using a MicroLab AT Plus (Hamilton Co., Reno, NV, USA) and Microplate Spectrophotometer (SpectraMax 340, Molecular Devices, Sunnyvale, CA, USA).

### Statistical Analysis

Descriptive statistics and identification of outliers, using the generalized extreme studentized deviation method [23] were performed. Exclusion of the two individuals identified as outliers (who give birth to very early preterm, < 32 weeks of completed gestational length) did not significantly alter any of our results and were retained in final models. In our final study population (N = 60) there were N = 12 (20%) individuals who delivered earlier than 37 weeks of completed gestational length. All urinary BPA concentrations (creatinine or specific gravity corrected and uncorrected) were log transformed in order to stabilize variance. Maternal and pregnancy characteristics were calculated and compared between those participants who delivered greater than 37 weeks and less than or equal to 37 weeks of gestation at time of delivery using Student's t-tests (2-tailed) and chi-square tests for continuous and categorical variables, respectively.

We calculated geometric means and distribution percentiles for uncorrected (μg/L), specific gravity corrected (μg/L), and creatinine corrected concentrations (μg/g creatinine) of BPA. The limit of detection (LOD) for BPA in a 0.1-mL urine sample was 0.4 μg/L. For concentrations below the LOD [N = 12 (20.0%)], an imputed value equal to one-half the LOD (0.2 μg/L) was used. BPA concentrations were corrected for urine dilution by specific gravity (SG) using the following formula: P_c _= P[(1.014 - 1)/SG - 1)], where P_c _is the SG-adjusted BPA concentration (ng/ml), 1.014 is the median SG value among the present study population, P is the measured BPA concentration, and SG is the specific gravity of the urine sample.

In preliminary crude analyses to assess whether women who delivered less than or equal to 37 weeks of gestation length had higher BPA concentrations than those who delivered after 37 weeks, geometric mean concentrations were compared between the two groups using t-tests (2-tailed). To take into account potential confounding variables, multivariable logistic regression was used to model the odds of delivering less than or equal to 37 weeks versus greater than 37 weeks of gestation. Variables considered in this analysis included maternal age, pre-pregnancy body mass index, third trimester urinary phthalate metabolites, parity, maternal education, marital status, and the infant's gender. Though we were unable to assess urinary or serum cotinine levels due to financial restraints, all of our participants were self-reported nonsmokers. Variables that did not differ between groups, and did not statistically confound (change in effect estimate of > 10%) in any of the models were not retained. The variables that did appreciably change (> 10%) the effect estimate in at least one of the models were included in all models for consistency.

A subset sensitivity analysis was performed in order to examine odds of delivering prematurely (< 37 weeks). In these additional models participants who delivered at 37 weeks of gestation were first included as controls and then excluded from the analysis. Data analysis and regression diagnostics was performed using SAS version 9.2 (SAS Institute Inc., Cary, NC, USA).

## Results

Maternal demographic characteristics for our final study population and for those non-smoking participants who delivered greater than 37 weeks, less than or equal to 37 weeks and preterm (< 37 weeks) are presented in Table 1. There were no significant differences in maternal age, maternal education, pre-pregnancy body mass index, parity, marital status, or infant sex when comparing term and preterm infants.

**Table 1:** Maternal and pregnancy characteristics for participants who delivered > 37 weeks, ≤ 37 weeks, and those participants who delivered preterm (< 37 weeks)

Characteristics	Participants who Delivered > 37 Weeks (N = 30)	Participants who Delivered ≤ 37 Weeks (N = 30)	Participants who Delivered Preterm (< 37 weeks; N = 12)
Years of Maternal Age [mean (SD)]	27.3 (5.9)	27.7 (6.2)	29.3 (5.9)

Years of Maternal Education [mean (SD)]	11.3 (2.5)	10.7 (3.6)	11.6 (2.9)

Prepregnancy BMI (kg/m^2^) [mean (SD)]	24.9 (4.4)	25.2 (3.8)	23.7 (4.2)

Parity [number (%)]			

First Child	10 (33)	10 (33)	4 (33)

Not First Child	20 (67)	20 (67)	8 (67)

Marital status [number (%)]			

Married	26 (87)	24 (80)	9 (75)

Single	4 (13)	6 (20)	3 (25)

Infant Sex [number (%)]			

Male	15 (50)	13 (43)	5 (42)

Female	15 (50)	17 (57)	7 (58)

** p-value < 0.05

BPA was detected in 80.0% (N = 48) of the samples with total concentrations ranging from 0.4 μg/L to 6.7 μg/L. The geometric mean and 95th percentile concentrations for our total study population were 1.52 μg/L (1.45 μg/L specific gravity adjusted) and 5.7 μg/L (4.36 μg/L specific gravity adjusted), respectively (Table 2.) Mothers who delivered less than or equal to 37 weeks had a geometric mean BPA concentration of 1.84 ± 1.86 μg/L (1.71 ± 1.57 μg/L SG adjusted) compared with mothers who gave birth after 37 weeks 0.97 ± 0.92 μg/L [1.20 ± 1.02 μg/L SG adjusted (Student's t-tests; p-value = 0.01 and 0.11 for unadjusted and SG-adjusted BPA concentrations, respectively)]. In addition, creatinine-adjusted BPA values between mothers who delivered less than or equal to 37 weeks and mothers who gave birth after 37 weeks were 2.20 μg/L and 1.70 μg/L, respectively (Student's t-test; p-value = 0.15.)

**Table 2:** Third Trimester Geometric Mean and Selected Percentiles of Uncorrected and Dilution Corrected Urinary Bisphenol-A Concentrations Among Mexican Women

BPA Metabolite	N	Geometric Mean	Percentile						
			
			10^th^	25th	50^th^	75^th^	90^th^	95^th^	Max
**All Participants**									

BPA (μg/L) uncorrected	60	1.52	0.20	0.55	0.95	1.80	3.15	5.70	6.70

BPA (μg/L) SG corrected	60	1.45	0.31	0.70	1.03	1.56	3.98	4.36	6.89

BPA (μg/g) Cr corrected	60	1.95	0.41	0.93	1.38	2.01	5.20	6.46	7.47

									

**Women who Delivered (> 37 weeks)**									

BPA (μg/L) uncorrected	30	0.97	0.20	0.20	0.80	1.10	2.05	3.40	3.90

BPA (μg/L) SG corrected	30	1.20	0.31	0.58	1.01	1.40	2.45	4.20	4.20

BPA (μg/g) Cr corrected	30	1.70	0.31	0.69	1.31	1.79	4.00	6.09	6.18

									

**Women who Delivered (≤ 37 weeks)**									

BPA (μg/L) uncorrected	30	1.84	0.35	0.70	1.05	2.20	5.70	6.40	6.70

BPA (μg/L) SG corrected	30	1.71	0.44	0.81	1.05	1.65	4.28	4.57	6.89

BPA (μg/g) Cr corrected	30	2.20	0.72	1.02	1.44	2.10	6.07	7.03	7.47

									

**Subset of Women who Delivered Preterm (< 37 weeks)**									

BPA (μg/L) uncorrected	12	2.18	0.80	0.80	1.15	2.65	6.20	6.70	6.70

BPA (μg/L) SG corrected	12	1.94	0.93	1.04	1.41	2.63	3.91	4.57	4.57

BPA (μg/g) Cr corrected	12	2.54	1.08	1.44	1.69	3.00	5.40	7.47	7.47

Crude and adjusted logistic regression models were fitted to explore the relationship between third trimester log transformed unadjusted, specific gravity adjusted, and creatinine adjusted BPA concentrations and the odds of delivering less than or equal to 37 weeks of gestation (Table 3.) All final multivariable models included covariates for maternal age, maternal education, infant gender and parity. The adjusted odds ratio of delivering less than or equal to 37 weeks in relation to specific gravity adjusted third trimester BPA concentration was 1.91 (95%CI 0.93, 3.91, p-value = 0.08). In a sensitivity analysis, crude logistic regression models were fitted to quantify this relationship in regards to delivering prematurely (< 37 weeks), acknowledging our small number of preterm cases (N = 12). In these models, in which we explored both including and then excluding women who delivered in the 37^th ^week in the control group, there were significantly increased odds of delivering prematurely with increasing specific gravity adjusted third trimester BPA concentrations (Table 4). Addition of third trimester urinary phthalate metabolites previously shown to be related to risk of prematurity in this population [24] as potential confounders didn't appreciably change the odds ratios in our analysis (data not shown).

**Table 3:** Unadjusted and Adjusted Odd Ratios for log transformed third trimester urinary bisphenol-A concentrations among women who delivered > 37 weeks compared to women who delivered ≤ 37 weeks

	**n**_**1**_	**n**_**2**_	Unadjusted	**Adjusted**^**a**^
			
			OR (95%CI)	OR (95%CI)
Log BPA (ug/L SG adjusted)	30	30	1.69 (0.88, 3.25)	1.91 (0.93, 3.91)

Log BPA (ug/g Cr)	30	30	1.58 (0.85, 2.97)	1.78 (0.90, 3.52)

^a ^Adjusted for: Maternal Age, Maternal Education, Parity, Sex

**Table 4:** Sensitivity Analysis of Unadjusted Odds Ratios for log transformed third trimester urinary bisphenol-A concentrations among women who delivered ≥ 37 weeks (and excluding those who delivered at 37 weeks) compared to women who delivered preterm (< 37 weeks)

			Unadjusted
	n_1_	n_2_	OR (95%CI)

**Including participants who delivered at 37 weeks as controls**			

Log BPA (ug/L SG adjusted)	48	12	2.50 (1.05, 5.96)
Log BPA (ug/g Cr)	48	12	2.11 (0.93, 4.80)

**Excluding participants who delivered at 37 weeks**			

Log BPA (ug/L SG adjusted)	30	12	3.24 (1.10, 9.60)
Log BPA (ug/g Cr)	30	12	2.36 (0.93, 6.00)

**Figure 1:**
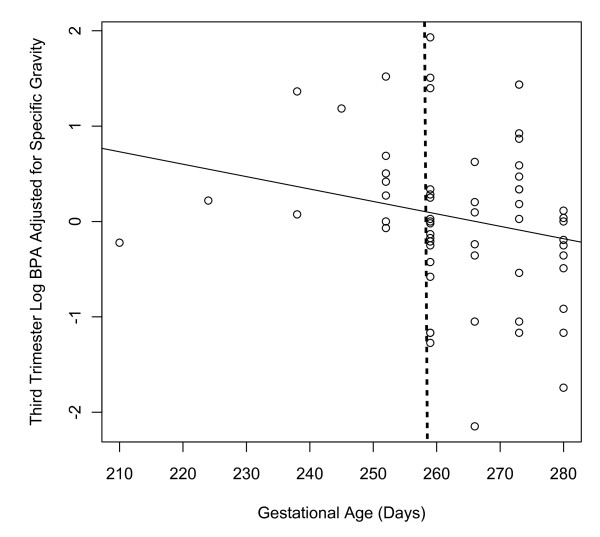
**Relationship between gestational length and log transformed third trimester urinary bisphenol-A concentrations among Mexican women (N = 60)**. The dotted line represents the 259 day (37 week) of gestation cutoff for preterm delivery.

The linear relationship between gestation length and log transformed, specific gravity adjusted, third trimester urinary BPA concentrations is presented in Figure 1. In multivariate linear regression models adjusted for maternal age, maternal education, parity, and infant sex, a 1-log increase in third trimester BPA concentration was associated with a 4.56 day (95%CI: -9.08, -0.04) decrease in gestation length (results not shown).

## Discussion

This is the first study to document measurable concentrations of BPA in the urine of a Mexican population. Furthermore, the present study suggests, based on a single spot urine sample collected during the third trimester, that women who delivered less than or equal to 37 weeks of gestation and prematurely (< 37 weeks) had higher concentrations of BPA in their urine compared to women delivering after 37 weeks.

Geometric mean urinary BPA concentrations (1.52 μg/L uncorrected, 1.95 μg/g Cr) in this pilot analysis from the ELEMENT study were very similar to concentrations among pregnant women in the Netherlands (1.1 μg/L uncorrected, 1.7 μg/g Cr) [7], New York City (1.3 μg/L uncorrected) [21], Cincinnati (2.0 μg/L uncorrected, 2.2 μg/g Cr) [25], and women in the general US population from NHANES 2003-2004 (2.4 μg/L, 2.8 μg/g Cr) [6]. The geometric mean concentration in our study was approximately one-half of that reported in a small (n = 10) subset of the Norwegian Mother and Child Cohort Study (4.5 μg/L uncorrected, 5.9 μg/g Cr) [11].

To date there has been limited human data on preterm birth, gestational length, and prenatal BPA exposure. In the Children's Environmental Health study, Wolff et al. found no association between BPA exposure during the third trimester and gestational length among 404 pregnant women in New York City [21]. In addition, a small scale study (N = 40) in Southeastern Michigan that measured BPA in blood of women at the time of delivery also found no difference in gestational length between women with blood BPA concentrations > 5 and ≤ 5 ng/mL[20]. However, unlike the present study, these studies were not designed to assess preterm birth in relation to BPA exposure. Other small-scale human studies of BPA exposure in pregnancy have reported increases in the risk of spontaneous abortion [26], sister chromatid exchanges [27], and abnormal fetal karyotype [28] with increased BPA concentrations. As far as we are aware, this is the first study to specifically assess the relationship between BPA exposure biomarker concentrations during pregnancy and preterm birth.

Preterm birth is likely a condition with multiple etiologies [29]. BPA may harm fetal growth and promote early parturition through various mechanisms, as it has been shown to disrupt a variety of biologic functions including steroid hormone synthesis and metabolism [30,31], peroxisome proliferation [32], cytokine networks [18], genotoxicity [33-35], and oxidative stress [36-40]. Low doses of BPA also induced apoptosis and increased output of matrix metalloproteinase-9, an enzyme associated with preterm birth, in ovarian granulosa cells in dose-dependent patterns [41,42]. Recently, it has been shown that human primary cytotrophoblast cells undergo a dose-dependent increase in TNF-α production and apoptosis with increasing environmentally relevant (0.0002 to 0.2 ug/mL) levels of BPA [18]. Supporting these initial results, Morck et. al. also demonstrated that similar levels of BPA exposure can induce cell death in a human choriocarcinoma cell line and increase secretion of β-hCG and caspase-3 cleavage in first trimester human chorionic villous explant cultures [17].

Our study has several limitations including its small sample size. Gestational length was estimated by date of maternally-recalled last menstrual period which may be an unreliable measure, varying as much as ± 7-21 days, depending on a host of factors including nutrition, physical activity, smoking, alcohol consumption, stress, and inter-pregnancy interval [43-46]. Because urinary BPA was only measured at a single time point in this study, the potential for measurement error exists since exposure is likely to be variable over time [47]. However, unless errors in last menstrual period recall were associated with BPA urinary concentrations and/or measurement error of BPA concentrations was systematic in relation to gestational age, one would expect that, on average, any bias in effect estimates would be toward the null.

## Conclusions

In conclusion, the results from this pilot analysis suggest that a group of pregnant women residing in Mexico City have similar urinary concentrations of BPA compared to women in the United States and other developed countries. In addition, based on the analysis of one spot urine sample collected during the third trimester of pregnancy, these results provide suggestive evidence that women who delivered less than or equal to 37 weeks of gestation and prematurely (< 37 weeks) have higher urinary concentrations of BPA compared to women who delivered greater than 37 weeks.

## List of Abbreviations

BPA: Bisphenol A; ELEMENT: Early Life Exposure in Mexico to ENvironmental Toxicants; NHANES: National Health and Nutrition Examination Survey; CDC: Centers for Disease Control and Prevention; SPE: solid-phase extraction; HPLC: high performance liquid chromatography; MS/MS: tandem mass spectrometry; Cr: creatinine; LOD: limit of detection; SG: specific gravity

## Competing interests

The authors declare that they have no competing interests.

## Authors' contributions

Authors contributed to the article as follows: DC conducted the literature review, designed and conducted the statistical analysis, and wrote the majority of the manuscript. JDM helped conduct the statistical analysis, wrote part of the manuscript, and provided critical feedback on the manuscript. HH designed the parent study, directed its implementation, and provided critical feedback on the manuscript. BNS helped to direct the statistical analysis/interpretation for the study and provided feedback on the Methods section. HLF helped to supervise the field activities and provided feedback on the manuscript. AMG helped supervise the field activities and primary data collection. GF provided feedback on the statistical analysis and manuscript. AMC supervised the analyses of samples for BPA and provided critical feedback on the manuscript. MMTR designed the parent study, directed its implementation, and provided feedback on the manuscript. All authors read and approved the final manuscript.
